# Influenza Vaccination of Romanian Medical Students and Resident Physicians—A Matter of Accessibility

**DOI:** 10.3390/vaccines11101551

**Published:** 2023-09-29

**Authors:** Ana Bălan, Simona Maria Ruță

**Affiliations:** 1 Faculty of Medicine, Carol Davila University of Medicine and Pharmacy, 050474 Bucharest, Romania; ana.balan@stud.umfcd.ro; 2 Stefan S. Nicolau Institute of Virology, 030304 Bucharest, Romania

**Keywords:** influenza vaccination, vaccine, healthcare students, vaccine hesitancy, vaccine accessibility

## Abstract

In Romania, influenza vaccination of healthcare professionals is recommended, but not mandatory. This study aims to investigate the attitudes and behaviors of medical students and resident physicians—the youngest healthcare professionals—towards influenza immunization, focusing on the barriers and facilitators, as well as on the impact of the COVID-19 pandemic. An anonymous online survey was conducted during the 2021/2022 influenza season, with responses from332 medical students and resident physicians. The majority (73.5%) were not vaccinated against influenza (68% of the students, 52.3% of the residents), although they were vaccinated against COVID-19 (94% students, 94.8% resident physicians) and believed that the pandemic positively influenced their attitude towards influenza vaccination. Vaccine accessibility (*p* < 0.001) and the necessity to pay for vaccination (*p* < 0.001) were identified as barriers in both groups, while lack of recommendation from a medical professional/teacher was significant only for students (*p* < 0.001). Forgetfulness and lack of prioritizations were the most cited reasons for not being vaccinated. These barriers could be diminished through proactive recommendation and simplification of the vaccination process, with accessible vaccination centers and implementation of vaccine reimbursement policies. Improved vaccination rates in young medical professionals are of the utmost importance both in their professional settings and as a model for the general population.

## 1. Introduction

Influenza vaccination protects against infection and severe complications (pneumonia, myocarditis, encephalitis) and reduces the number of hospitalized cases and deaths attributable to influenza. The WHO Global influenza strategy for 2019–2030 underlines the positive cost-effectiveness ratios (ranging from USD 10,000 to USE 50,000 per outcome) of vaccination versus non-vaccination [1,2]. Consequently, there are strong recommendations at the global, regional, and national levels for annual vaccination of risk groups (children aged 6 months–5 years, pregnant people, elderly people, persons with specific comorbidities) and healthcare workers, who are at high risk of infection in the professional setting and important players in the chain of transmission. Healthcare workers are often the source of influenza outbreaks, at both the hospital and the community level [3]. A meta-analysis published in 2011 reported influenza infection rates of 16–22% among unvaccinated healthcare workers between 1950 and 2010 [4]. During outbreaks of nosocomial influenza infection, 20–50% of healthcare workers can be affected and can further transmit the infection to vulnerable patients, who are at increased risk for influenza direct complications, as well as for exacerbations of preexisting heart or respiratory diseases [2]. Conversely, immunization of healthcare workers can protect the most at risk patients, although there is a lack of data on the impact on influenza-related complications for patients in long-term care facilities [5]. According to a CDC analysis, during the 2021–2022 influenza season, the overall vaccination rate of healthcare personnel was 79.9%, with important differences according to the education level (more than 90% among clinical personnel with higher education -physicians, pharmacists, nurse practitioners) versus 68–75% among other employees with lower education level -clinical assistant/aides and non-clinical staff, who reach only 68–75%). However, the vaccination rate was much lower (48.1%) for personnel working in clinics where vaccination was not mandatory, offered, or promoted [6]. Thus, natural compliance with this method of influenza prevention is not very high.

The COVID-19 pandemic led to unprecedentedly restricted social measures (movement restrictions, social distancing, school closures, remote working, facial masks use) that decreased transmission of all respiratory viruses, including influenza [7]. As a result, the number of influenza cases was very low in 2020–2021, with only 168 cases reported in Europe [8]. In addition, worldwide, there have been reports suggesting a beneficial effect of the COVID-19 pandemic on influenza vaccination uptake. For example, a study conducted in Italy on healthcare workers over a 3-year period showed a 230% increase in the vaccination rate in the 2020/2021 season (39% versus 11.9% in the 2019/2020 season and 10.8% in the 2018/2019 season) [9].

In Romania, a country with a population of around 19 million, the influenza vaccination rate—as of pre-pandemic 2017/2018 data—was very low, ranging from 7.4% to 14.9% for elderly people, from 2.6% to 4.2% for pregnant people, and 17.8% for those included in clinical risk categories [10]. For healthcare workers, the vaccination rate was 34% during the same season [11], while the estimated financial burden of the influenza was 0.74% of the national health budget [12]. No data are available on the vaccination rate of medical students, the youngest healthcare workers, who spend most of their daily training in hospitals, in direct contact with vulnerable patients, and who have high mobility, attending multiple clinical internships during the year, thus playing an important role in the transmission of respiratory infections.

The aim of this study is to investigate the rate of influenza vaccination in healthcare students and resident physicians in Romania during the 2021/2022 season, as well as the factors influencing their vaccination behavior and the impact of the COVID-19 pandemic on this issue. 

## 2. Materials and Methods

A descriptive and observational cross-sectional survey was conducted to assess the attitudes towards influenza vaccination among young medical professionals (healthcare students, who, although not formally employed, spend a considerable amount of their training in hospital settings in direct contact with patients and medical residents). Data were collected through two electronic questionnaires utilizing Google Forms, the first questionnaire being designed for students, while the second was dedicated to resident doctors. Each questionnaire consisted of 31 items, categorized into four sections: general data (5 items), behaviors (6 items), attitudes (15 items), and knowledge (5 items). The questions included multiple-choice, single-choice, Likert scale, and open response formats. 

Respondents remained anonymous, email addresses were not collected, and the questionnaire was set to accept a single answer per email address to prevent duplication. The inclusion criteria for participants were either being a student at Carol Davila University of Medicine and Pharmacy, verified through the institutional address, or being a resident doctor (medical residency in Romania ranges from 4 to 6 years of training). The institutional address was not a requirement for resident doctors due to their association with residency coordinators at other universities or the absence of an institutional address. The only exclusion criterion was disagreement with the handling of personal data. To reach the target audience, the two questionnaires were disseminated through communication networks like Whatsapp, Facebook, and Instagram, specifically targeting groups composed of students and resident doctors, during February–March 2022. Approval from the University’s ethics committee was obtained to communicate the results in the form of scientific articles and presentations. 

The variables measured were categorized into four groups. The first category pertained to demographic data, including gender, age, background, year of study (for students), year of training (for residents), faculty (for students), and specialty (for residents). The second section focused on behaviors and consists of six nominal variables. The third section explored attitudes, which were assessed using eight nominal variables and seven ordinal variables. When asked about their reasons for not being vaccinated against influenza, respondents were given a choice from a list of predefined answers, with indications to choose any number of reasons and/or to add supplementary motives if needed. The final category of items related to knowledge, comprising three nominal variables and two ordinal variables.

Using the visualization tools provided by Google Forms, a descriptive analysis of the data was performed reporting on gender, age, faculty/specialty, year of study/year of training, background, and general data on respondents’ attitudes, behaviors, and knowledge about influenza vaccination in the context of the COVID-19 pandemic. For analytical statistics, the free statistical processing software Jamovi 2.3.26 was used to test the association between knowledge, behaviors, attitudes and acceptability, access to influenza vaccine, and main reasons for vaccine hesitancy. The statistical tests used are chi-square for the student group and Fisher’s exact test for the resident group due to the small sample size. To analyze the differences between the two groups of respondents, Barnard’s test was used for the nominal variables. The alpha value of the p value is set at 0.05. The sample size was calculated using the formula n = (Z^2^ × p × (1 − p))/E^2^, where n is the total number of respondents, in this case 332; Z is the z-score associated with the desired confidence level, in this case 95%, generating Z = 1.96; p is the estimated proportion of the population with the characteristic of interest; and E is the margin of error expressed as a decimal.

## 3. Results

### 3.1. The Study Population 

The entire study population consisted of 332 respondents: 285 (85.8%) students and 47 medical residents (14.2%). The respondents were mostly female (77.4%), coming from urban regions (89.4%) and with a mean age of 22 years. The majority of the students (90.1%) were students at the medical school, while the rest were studying nursing and midwifery (5.4%), dentistry (0.7%), and pharmacy (1%). As for the year of study, the students were grouped in descending order as follows: 30.2% in the second year, 24.9% in the fifth year, 17.9% in the third year, 11.9% in the fourth year, 10.2% in the sixth year, and 4.9% in the first year. Medical residency in Romania ranges from 4 to 6 years of training. The resident respondents were mostly in their first years of training: 48.9% in the first year, 23.4% in the third year, 21.3% in the second year, 4.2% in the fourth year and only one in the fifth year. They came from diverse specialties (psychiatry, pediatrics, medical imaging, neurology), and none were specializing in infectious diseases, epidemiology, or microbiology.

### 3.2. Vaccination Rate

Out of the 332 respondents, only 77 (23.2%) were vaccinated against influenza. Of the vaccinated, 44 (57.1%) opted for the tetravalent inactivated vaccine, 28 (36.4%) did not know the type of vaccine they were immunized with, and 5 (6.5%) chose the live attenuated tetravalent vaccine. The majority of the respondents (73.5%) were not vaccinated (68% of the students, 52.3% of the residents); out of these, 36 (10.8%) identified themselves as undecided. The year of study or training did not influence the vaccination status in either group. 

On the contrary, the majority of the respondents were immunized against COVID-19 (94% students, 94.8% resident physicians)—207 (62.3%) have received three SARS-CoV-2 vaccines doses, 107 (32.2%) have received two doses, and only 18 (5.5%) were not vaccinated. When asked if they consider themselves at a higher risk of SARS-CoV-2 infection than the general population, most of them (71.1%) answered affirmatively. Paradoxically, most respondents (54.2%) felt that the COVID-19 pandemic made them more likely to consider getting vaccinated against seasonal influenza. 

### 3.3. Knowledge about Influenza

Overall, the respondents demonstrated accurate identification of high-risk groups, including those aged 15–64 with comorbidities (84.9%), individuals over 65 (74%), children under 2 years old (46.4%), and pregnant people (40%). However, 66 (19.8%) respondents incorrectly selected the option of individuals aged 15–64 with no comorbidities as a high-risk group. When asked to identify influenza complications, 96.6% respondents correctly identified pneumonia, 60.5% correctly identified encephalitis, and 56% correctly identified myocarditis. However, 35.8% respondents mistakenly identified gastroenteritis, despite this not being a typical complication of influenza. 

### 3.4. Factors Influencing the Vaccination Behavior

Forgetfulness and lack of prioritization were the two most-cited reasons for those who did not take up the recommendation for vaccination, followed by a perceived lack of personal risk for a severe infection, while only two respondents (0.06%) were opposed to vaccines in general. 

Respondents were asked to rate the perceived level of safety of influenza vaccines using a Likert scale from 1 (lowest level of safety) to 5 (highest level of safety). For simplicity, three groups have been created: vaccine considered safe (for those selecting 4 or 5 on the Likert scale), neutral stance (for those selecting 3 on the Likert scale) and vaccine considered unsafe (for those selecting 1 and 2 on the Likert scale). The majority of the respondents—298 (89.7%)—considered the vaccine safe, 31 (9.3%) had a neutral position, while only 3 (1%) considered it unsafe. 

The fear of allergic reactions was assessed using a 5-point Lickert scale, ranging from 1 (lowest level of concern) to 5 (highest level of concern), grouping the answers in a similar way—no/low concern (those answering 1 and 2), neutral (those selecting 3), and concerned (those selecting 4 and 5). Most respondents (80.4%) reported a low level of concern regarding potential allergic reactions, 16% were neutral, and 3.6% considered themselves concerned. Very similar proportions were recorded in terms of fear of adverse reactions and long-term side effects, evaluated with a similar 5-point Lickert scale. 

In terms of vaccine effectiveness perception, 267 respondents (79.6%) considered the flu vaccine to be effective, 46 (16.1%) had a neutral attitude, and 12 (4.3%) considered the vaccine ineffective.

Most respondents did not consider the vaccine cost a barrier, but 76.2% would get vaccinated if the flu vaccine was available free of charge at vaccination centers, similar to the COVID-19 vaccines. When asked if they would get vaccinated at such a center, but for a fee, the proportion of those who said yes was lower (60.2%). 

Only 23.8% of the respondents reported receiving a vaccination recommendation from their teachers or residency coordinator. 

A majority of 85.5% of respondents were aware of the recommendation for annual vaccination and correctly answered that the flu vaccine is updated annually due to antigenic drift of influenza viruses, although other reasons factor into this process, such as antigenic shift and the evolving epidemiology of the different circulating strains of the virus. 

When asked if the type of vaccine or the technology used for vaccine manufacturing was important for their decision to be vaccinated, the majority of respondents—218 (67.7%)—answered that they were not influenced by it, while 69 (21.4%) would rather be vaccinated with a flu vaccine produced by another technology (e.g., mRNA, recombinant protein or viral vector), and 35 (10.9%) would not chose another type of vaccine. An important proportion of the respondents (46.3%) were not aware about the availability of a live attenuated influenza vaccine administered intranasally to children and young adults, 47.6% knew about this formulation, and only 6.1% wrongly believed such a vaccine did not exist. 

### 3.5. Comparison between Vaccinated and Unvaccinated Respondents

To investigate factors influencing the decision to vaccinate against influenza we compared the responses of vaccinated and unvaccinated subjects (Table 1). 

The necessity to pay for vaccination (*p* < 0.001) and accessibility (*p* < 0.001), assessed as the availability for free vaccine uptake in immunization centers, similar to those established during the pandemic for COVID-19, were identified as barriers for influenza vaccination in all respondents.

The lack of recommendation from a medical teacher or residency supervisor was found to be significant (*p* < 0.001); although, in a separate analysis, when comparing students and medical residents, this was true only for the students (students *p* < 0.001, medical residents *p*—0.23). 

Uptake of the complete course of three doses of COVID-19 vaccine was also a significant factor associated with influenza vaccination status (*p* < 0.001), as was respondents’ higher perceived risk for infection (*p*—0.018). In contrast, in the analysis of the whole group, the third-most-voted reason for vaccine avoidance was the perceived lack of personal risk of disease (“I never got influenza, influenza is not severe for me”), which may be explained by an acknowledged risk of infection and transmission, but a dismissal of the personal risk of a serious illness. 

On a Lickert scale, fear of adverse events was associated with non-vaccination for short-term side events (*p* = 0.003), allergic reactions (*p* = 0.017), or adverse reactions on the long term (*p* = 0.026).

## 4. Discussions 

### 4.1. Influenza Vaccination Rates

Determining the influenza vaccination rate among healthcare students and resident physicians is a challenging task, due to the limited availability of systematic reviews on this topic. 

Both prior to the COVID-19 pandemic and currently, the global influenza immunization rate among students has typically ranged between 11.9% [13] and 71.2% [14], with some exceptions. However, as shown in the present study, in Romania, the influenza vaccination rate in the 2021 season was 23.2% (21% for medical students and 35.4% for resident physicians). A previous study [15] conducted during the COVID-19 pandemic period (2020/2021 season) at the Carol Davila University of Medicine and Pharmacy on 1581 healthcare students reported a flu vaccination rate of 30.3%. 

These data are consistent with those reported in other studies on medical students. In a 2012 German survey [16], which included 264 medical student respondents, the vaccination rate was 12.9%. In Italy, a questionnaire [17] conducted during the 2017–2018 period among 3,000 health students revealed that 11.2% had received the influenza vaccine in the previous season, but approximately one third of all respondents expressed their intention to get vaccinated in the future. However, previous data from Italy [13] indicated that the vaccination rate for resident doctors was on a decreasing trend: from 21.7% in the 2008/2009 season to 11.9% in the 2011/2012 season. Likewise, a survey of 341 students in Saudi Arabia [18] reported a 30.7% rate in the current season (2020/2021), while 80.4% of respondents claimed to have been vaccinated in previous seasons. Likewise, medical students in Cyprus [19] had a vaccination rate of 20.1% in the 2020/2021 season, but indicated that 50.8% had been vaccinated in previous seasons. A higher rate of influenza vaccination was reported in a Canadian study [14] involving 300 healthcare students and covering two influenza seasons (2014–2016) prior to the pandemic: 85.4% in the 2014/2015 season and averaging 71.2% across both seasons. It is important to note here that vaccination was mandatory for the population included in this particular study. 

Although most respondents in our study reported that the COVID-19 pandemic increased their awareness and interest for influenza vaccination, the actual vaccination rate was comparatively low. The combined effects of a perceived low risk of influenza, fueled by the low number of influenza cases during the pandemic, and of an increasing vaccination hesitancy may explain this discrepancy, a result that was also reported in several other countries. For example, findings from a survey [20] conducted in China in 2022 involving over 2000 students showed a hesitancy rate of 44.7% regarding influenza vaccination.

These studies, as well as the present one, highlight a consistent trend: while there is a positive intention to get vaccinated against influenza, numerous barriers exist that hinder the realization of this intention. Within our studied population, the accessibility and necessity to pay for vaccination were identified as obstacles to vaccination. However, in the case of healthcare workers examined in the afore mentioned studies, these factors were not linked to their intention to get vaccinated. A survey of health workers in Jordan [21] on influenza vaccination reveals a similar perception among health professionals, who agree that they are at higher risk of infection and acknowledge the potential severe complications of influenza, but this does not influence their intention to vaccinate. This study concluded that providing free vaccines may not have a substantial impact, and instead, interventions should be targeted towards individual risk factors. 

It is worth mentioning that in the present survey, the question on influenza immunization status in the 2020/2021 season had three possible answers: Yes, No, and Not yet. As the questionnaire was distributed at the end of the influenza season, in the analysis, the undecided minority (10.8% of all respondents) was included in the group of unvaccinated, as this would only reflect a weak intention to be vaccinated in the last months of the pandemic season, or perhaps a more socially desirable way of saying No. When performing the same statistical analysis on the three initial vaccination groups—vaccinated, unvaccinated, and undecided—statistically relevant results remain the same, with the exception of adverse reactions that did not reach a statistical threshold. This might suggest that fear of unwanted reactions to the flu shot could be the reason for postponing vaccination in the undecided group.

### 4.2. Cost and Accessibility

In our study, when questioned about their reasons to skip vaccination, only 8 (0.02%) respondents selected the actual cost as an impediment. As such, the low-income levels among students cannot represent a barrier for influenza vaccination. Instead, forgetfulness, lack of prioritization, or lack of encouragement to get vaccinated were reasons selected significantly more frequently. This may be due both to the relatively low cost of influenza vaccine (around EUR 15) and to a multi-step process of accessing vaccination, that requires a visit to the epidemiologic center of the hospital in which students and residents are in practice, in order to be eligible for free vaccination. 

Concordantly, a study conducted in China [22] among students from various fields found that their monthly living expenses did not influence their decision to vaccinate. In another prospective study involving medical students in China [20], both vaccine inaccessibility and inconvenience were identified as barriers before the pandemic in 2019 and during the pandemic in 2021 to a similar extent. Considering the weaker association between vaccine cost and vaccination intention in the previously mentioned studies, it can be argued that although the necessity to pay might represent a barrier, improving vaccine accessibility would be more effective than offering vaccines for free. This may be especially important in reducing the intention–behavior gap [9], as the direct payment of the vaccine is one of the last steps in a notable process starting with the awareness of the need to be immunized against influenza, followed by the intention, and then acting on this intention.

### 4.3. Teacher or Supervisor Recommendation 

In our study, the lack of recommendation from either teachers or supervisors had a negative impact on influenza vaccination, being listed as the second most common reason for not being vaccinated. The most common reason, however, was that despite the recommendation, the vaccine was not a priority or that the respondent had forgotten about vaccination. A Canadian study [14] also assessed the impact of recommendation by a teacher or tutor, with a significant proportion of vaccinated students citing this as a secondary reason for vaccination, while individual protection, patient protection, and the obligation to vaccinate were among the main reasons. In the same survey, forgetfulness or lack of time was most often cited as the main reason for not vaccinating.

### 4.4. Limitations of the Study

Although the target audience of this questionnaire is part of the digitally native generation, the study has a slightly smaller sample size than expected. This may be due in part to questionnaires fatigue, a phenomenon that might be caused by the self-reported vast number of online surveys reaching students in recent years through formal and informal channels. The smallest size was for the resident physicians, most in their first years of training, who may be subjected to selection bias, due to the lack of well-established communication channels with the entire young doctors’ community. Nevertheless, the entire respondent sample size reached statistical significance and a separate analysis of the two categories did not reveal changes in the significant results.

## 5. Conclusions

In an online survey conducted during 2021/2022 influenza season on medical students and resident physicians in Romania, most respondents were found to not be vaccinated against influenza, although most of them perceived themselves to be at a higher risk of infection compared to the general population. Nevertheless, a large majority of respondents are vaccinated against SARS-CoV-2 and feel that the pandemic has positively influenced their decision to get the influenza shot. The vaccine is perceived as safe and effective by most respondents, despite an alleged concern caused by adverse reactions or allergic reactions in those who are unvaccinated.

Respondents were not found to be opposed to vaccination in general; forgetfulness, lack of prioritization, and perceived difficult access were the main reasons for not being vaccinated. Accessibility of the vaccine is a barrier to vaccination to a greater extent than its actual cost, although both factors have a significant influence on vaccination. In this study, lack of encouragement from teachers negatively influenced influenza vaccination rates. Simplification of the vaccination process, including establishment of accessible vaccination centers, similar to the ones available during the pandemic for SARS-CoV-2 vaccination, and implementation of vaccine reimbursement policies may improve vaccine adherence. 

## Figures and Tables

**Table 1:** Factors influencing the decision to vaccinate against influenza.

Factor	Overall		Vaccinated against Influenza		Unvaccinated		*p*
	(n = 332)		(n = 77)		(n = 255)		
Recommended by teacher/coordinator							<0.001
Yes	79	23.8%	32	41.5%	45	17.5%	
No	253	76.2%	47	61%	208	81.5%	
Vaccinated against COVID-19							<0.001
Yes, 3 doses	207	62.3%	64	83.1%	143	56%	
Yes, 2 doses	107	32.2%	12	15.6%	95	37.3%	
No	18	5.4%	1	1.3%	17	6.7%	
Perceived themselves at greater risk for influenza than the general population							0.018
Yes	236	71.1%	63	81.8%	173	67.8%	
No	96	28.9%	14	18.2%	82	32.2%	
Fear of allergic reaction							0.017
1	200	60.2%	57	74%	143	56.1%	
2	76	22.9%	16	20.8%	60	23.5%	
3	39	11.7%	2	2.6%	37	14.5%	
4	12	3.6%	1	1.3%	11	4.3%	
5	5	1.5%	1	1.3%	4	1.6%	
Fear of adverse reaction							0.003
1	162	48.8%	48	62.3%	114	44.7%	
2	101	30.4%	25	32.5%	76	29.8%	
3	43	13%	2	2.6%	41	16.1%	
4	19	5.7%	1	1.3%	18	7.1%	
5	7	2.1%	1	1.3%	6	2.4%	
Fear of long-term adverse reactions							0.026
1	228	68.7%	64	83.1%	164	64.3%	
2	66	19.9%	10	13%	56	22%	
3	24	7.2%	1	1.3%	23	9%	
4	9	2.7%	1	1.3%	8	3.1%	
5	5	1.5%	1	1.3%	4	1.6%	
Would take-up the vaccine if free							<0.001
Yes	253	76.2%	72	93.5%	181	71%	
No	79	23.8%	5	6.5%	74	29%	
Would take-up the vaccine if available in designated centers for a fee							<0.001
Yes	200	60.2%	68	88.3%	132	51.8%	
No	132	39.8%	9	11.7%	123	48.2%	

## Data Availability

All data presented are available upon request from the corresponding author (S.M.R.).
